# Experimental Evaluation of Concrete Blended with Eco-Friendly Bio-Sulfur as a Cement Replacement Material

**DOI:** 10.3390/ma17236016

**Published:** 2024-12-09

**Authors:** Wonchang Kim, Taehyung Kim, Taegyu Lee

**Affiliations:** Department of Fire and Disaster Prevention, Semyung University, Jecheon-si 27136, Choongbuk, Republic of Korea; firesafety9505@gmail.com (W.K.); 174cm@kakao.com (T.K.)

**Keywords:** concrete, bio-sulfur, eco-friendly, ultrasonic pulse velocity

## Abstract

Bio-sulfur (BS), extracted from landfill bio-gas via microbial methods, was examined herein as a potential cement replacement material. The study developed five modified BS variants through limestone incorporation processes (sulfur-to-limestone ratios of 1:0.5, 1:1, 1:1.5, 1:3, and 1:5). The study revealed that modified BS with higher limestone ratios demonstrates significant workability and strength reductions of over 50% with increased content, leading to the adoption of a sulfur-to-limestone ratio of 1:1. The concrete specimens exhibited compressive strength improvements of up to 12% with increased BS content, while the UPV showed proportional increases with increased BS content that remained independent of the water/binder (W/B) ratio. Statistical analysis confirmed significance with *p*-values below 0.05. XRD analysis identified initial cement hydrate peaks at 3 d that evolved into distinct Mg-S hydrate and Ca-Al-S hydrate formations in the BS-containing specimens by 28 d.

## 1. Introduction

With growing emphasis on eco-friendly technology development for greenhouse gas reduction and carbon neutrality, industrial waste utilization and carbon dioxide emission reduction are receiving increasing attention in existing manufacturing processes [1,2,3]. The construction industry in particular, as the largest global emitter of carbon dioxide, is showing great interest in reducing cement usage, and research has been actively conducted on various industrial by-products and previously unused natural resources to this end [4,5]. Notably, the global sulfur concrete market shows steady growth, reaching approximately USD 400 million in 2023, with experts forecasting its expansion to USD 814.74 million (a projected annual growth rate of 9.3% from 2024 to 2031) [6,7].

Sulfur dioxide (SO_2_), resulting from the production of natural gas and petrochemical products, is one of the main causes of air pollution [8]. With ongoing electricity generation, industrial boilers, oil refining, and the development of other industries, concerns regarding environmental pollution are growing, bringing to the forefront the topic of sulfur emission reduction [9,10,11]. Several North American, European, and Asian nations have implemented strict sulfur emission regulations, driving continuous research into sulfur recovery and utilization technologies. Although approximately 54% of global sulfur production serves agricultural fertilizer needs, particularly in agriculture-dependent Asian nations, current utilization rates remain insufficient. Despite additional applications in cosmetics, textiles, and pharmaceuticals, excess sulfur remains, requiring landfill disposal. Consequently, the development of methods to utilize sulfur as a construction material offers a viable solution for large-scale processing and is supported by the extensive literature on sulfur concrete applications.

Fediuk et al. [12] documented sulfur-based concrete characteristics, highlighting early strength development as a key feature, in contrast to conventional cement. Zheng et al. [13] observed earlier peak hydration heat in sulfur powder mixtures compared to pure cement specimens. This was attributed to the role of sulfur in facilitating calcium ion dissolution and precipitation in pore solutions while promoting clinker mineral dissolution through reduced nucleation barriers [13]. Furthermore, studies by Fediuk et al. [12], Zheng et al. [13], and Stel’makh et al. [14] demonstrated enhanced strength development through sulfur’s filler effect when compared to pure cement. Shim et al. [15] reported strength improvements through CaSO_4_ formation resulting from sulfur–calcium cement reactions. Based on these research findings, utilizing sulfur as a cement replacement material in construction is expected to provide significant advantages.

While previous research focused on employing powdered petroleum sulfur as a construction material, recent studies have reported instances of concrete applications utilizing the dicyclopentadiene modification method for refined petroleum sulfur [16,17,18,19,20,21]. This approach addresses both environmental concerns arising from heat and gas emissions during powder production and economic issues related to continuous manufacturing and processing. Since sulfur is insoluble in water, various attempts have been made to manage it at room temperature by converting it into a liquid or water-soluble form by dissolving it at temperatures between 119 and 159 °C. However, petroleum sulfur remains solid at room temperature and requires remelting at temperatures above 80 °C for reuse, thus creating practical limitations for construction site applications. In contrast, bio-sulfur (BS) produced through sulfur-oxidizing bacterial metabolism can maintain a liquid state at room temperature and shows hydrophilic properties that can be advantageous for construction material applications and quality management. Consequently, to develop a modified sulfur with enhanced usability and safety characteristics, this study explored the use of BS produced through ‘microbial processing’. In addition, an indirect evaluation method using ultrasonic pulse velocity (UPV) among non-destructive methods has been proposed to predict the strength of conventional concrete, but there is no study on the strength prediction model of concrete mixed with BS. Therefore, a strength prediction model is presented to predict the strength development of concrete mixed with BS during the construction stage and to evaluate the time of formwork demolding.

In this study, in order to evaluate the mechanical and chemical properties of bio-sulfur concrete (BSC), the best modified BS was selected based on the material quality and stability and mortar test results, and compressive strength, UPV, and XRD analyses were performed by mixing it into concrete. In addition, a strength prediction model was proposed through the regression analysis of UPV and compressive strength and compared with the demolding strength of the formwork specified in ACI 347R [22] and BS 8010 [23].

## 2. Bio-Sulfur

### 2.1. Production Process

**Figure 1:**
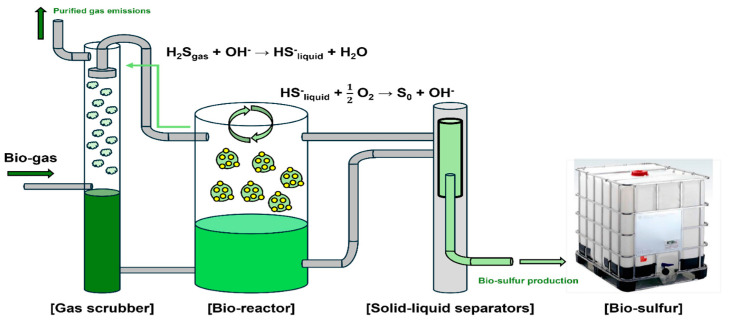
Process for the production of BS.

Figure 1 shows the production of BS through microbial processing. Hydrogen sulfide (H_2_S) contained in bio-gas and landfill gas from waste disposal sites requires mandatory removal because of its odor, corrosive nature, and contribution to air pollution. Therefore, technology for separating hydrogen sulfide into hydrogen and sulfur components is required for sulfur utilization. Recently, pretreatment methods employing sulfur-oxidizing bacteria have gained wider adoption and implementation, with this study incorporating the THIOPAQ process developed by Dutch company P [24].

**Figure 2:**
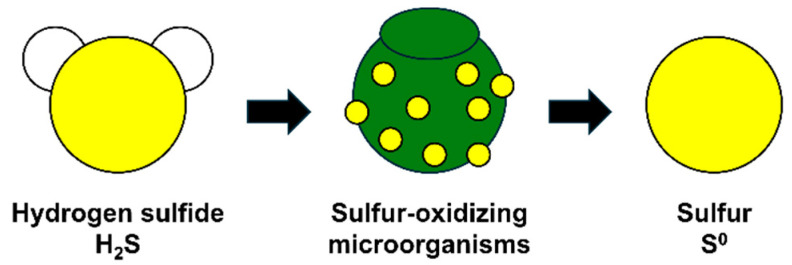
BS production process using a bio-reactor.

Initially, bio-gas hydrogen sulfide is absorbed in a gas scrubber, then transformed into elemental sulfur (S0) in a bio-reactor containing sulfur-oxidizing bacteria (see Figure 2), with BS produced through subsequent gravity concentration and centrifugation processes. BS produced through microbial processing exhibits hydrophilic characteristics, a fine particulate structure, and mild alkalinity owing to the aqueous microbial metabolic processes used in its production. Traditional sulfur produced through petrochemical processes remains solid at room temperature and demonstrates hydrophobic properties, presenting significant challenges for on-site material quality control and stability. In contrast, BS produced through sulfur-oxidizing bacterial metabolism can maintain a liquid state at room temperature and shows hydrophilic properties, suggesting advantages for construction material applications and quality management.

### 2.2. Modification

BS melts within a temperature range of 100 ± 10 °C and, in its molten state, transforms from a solid to a liquid state through a reaction with calcium ions, as shown in Equation (1).

CaO(solid state) + S(solid state) + H_2_O → Ca(OH)_2_ + S(liquid state) (1)

The BS in the liquid state further reacts with residual Ca(OH)_2_ to form calcium–sulfur compounds, with the simultaneous presence of products from Equations (1)–(3) when the reactions are incomplete.

3Ca(OH)_2_ + 12S → 2CaS_5_ + CaS_2_O_3_ + 3H_2_O (2)

3CaO + 12S + CaSO_4_(solid state) + H_2_O → 2CaS_5_ + CaS_2_O_3_ + CaSO_4_(solid state) + H_2_O (3)

Modified BS application enables calcium–sulfur compound formation, such as CaS_5_ and CaS_2_O_3_, from BS and limestone interactions, with Ca_2_^+^ ion leaching and ettringite formation through 3CaO·Al_2_O_3_ reactions during cement hydration. This process is expected to enhance matrix densification, thus improving durability by reducing external harmful ion penetration and diffusion [25]. Furthermore, unreacted calcium–sulfur compounds within the cement matrix voids are anticipated to both reduce harmful ion infiltration and enhance strength development.

## 3. Experimental Procedure

### 3.1. Experimental Plan

Table 1 outlines the study’s experimental design. To determine the optimal modified BS ratio (sulfur to limestone), mortar specimens were prepared and tested, with the highest-performing BS selected for concrete implementation. The mortar incorporated six different proportions of BS (0%, 3%, 6%, 9%, 12%, and 15%), and the concrete included three proportions of BS (0%, 5%, and 7%). Five variations of the modified BS were evaluated (sulfur to limestone = 1:1.5, 1:3, 1:5, 1:1, and 1:0.5). The mortar evaluation used the day 3 and day 7 results to determine the appropriate BS proportions for concrete mixing. Concrete target strengths were established at 5000 psi (35 MPa) and 6500 psi (45 MPa), utilizing W/B ratios of 0.40 and 0.32, respectively, to achieve those strengths. The specimens underwent water curing and constant temperature/humidity conditions, with mechanical property assessments at 3, 7, and 28 d. The study evaluated the fresh concrete slump and hardened concrete properties, including unit weight, compressive strength, ultrasonic pulse velocity (UPV), and X-ray diffraction (XRD), proposing a strength prediction model through compressive strength and UPV regression analyses. The compressive strength of the concrete was evaluated using a UTM with a maximum capacity of 1000 kN. The UPV measurements were performed utilizing Ultracons-170 equipment with 60 kHz P-wave transducers. To analyze the crystalline structure and composition of the concrete, XRD was performed using a Bruker (D2 Phaser, Billerica, MA, USA).

### 3.2. Materials

**Figure 3:**
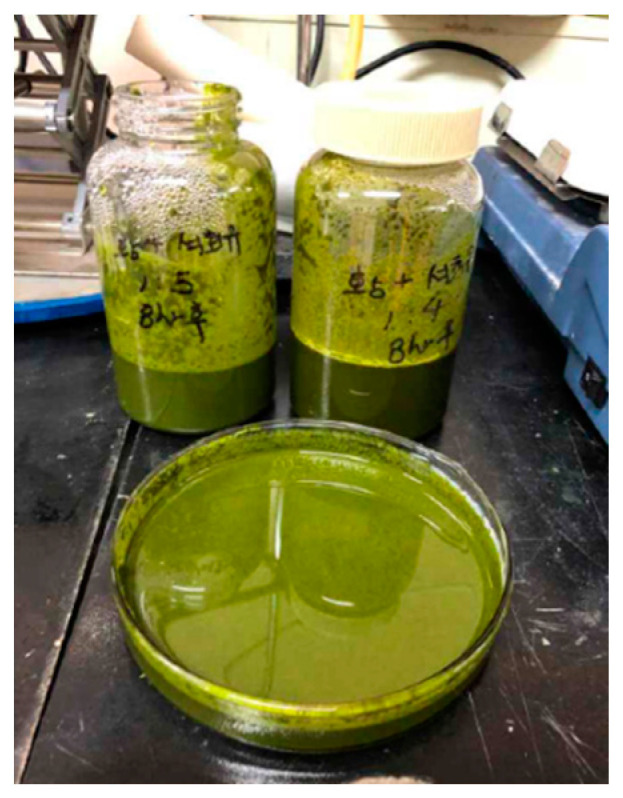
Bio-sulfur.

Table 2 presents the physical properties of the materials utilized in this research. The BS has a lower density (1.41 g/cm^3^) than ordinary Portland cement (OPC). When modified with limestone, the BS exhibited a blue-green slurry state, as shown in Figure 3. The chemical composition of the OPC and BS is detailed in Table 3. For concrete mixing, crushed granite was utilized as the coarse aggregate, and river sand was utilized as the fine aggregate.

### 3.3. Concrete Mix Proportion and Test Method

The mortar mixtures in this study were prepared according to the ISO 679 standard [26], using a binder/water/fine aggregate ratio of 1:0.5:3, with five types of BS incorporated at six different proportions (0%, 3%, 6%, 9%, 12%, and 15%). After mixing, the specimens were cured at 20 ± 2 °C for 24 h, followed by water curing up to 28 d.

Table 4 shows the concrete mix proportions. The numbers after Plain and BSC refer to W/B. The BS selected for concrete mixing was chosen based on mortar slump and strength evaluations; the type exhibiting the highest material stability and quality was selected. Although the modified BS existed in the liquid state at room temperature, some undissolved sulfur and limestone remained present. Therefore, aggregates and cement were initially mixed and then combined with water to ensure an even distribution of precipitated powder in the BS. Concrete mixing and specimen preparation followed the ASTM C31 standards [27], with specimens cured at 20 ± 2 °C for 24 h, followed by water curing for up to 28 d.

The concrete slump was tested according to ASTM C143/C143M-12 [28] and compressive strength according to ASTM C39/C39M-21 [29]. Before the specimen was placed in the UTM machine and loaded, the cross-section was polished using a polishing machine to ensure even load distribution. The UPV testing followed the ASTM C597-22 protocol [30], applying vacuum grease to transducers to ensure proper contact with the concrete surfaces. Following compressive strength and UPV evaluations, a concrete strength prediction model was developed through regression analysis for indirect strength assessment using UPV. Additionally, an XRD analysis was conducted to qualitatively evaluate the reaction products of cement and BS.

## 4. Results and Discussion

### 4.1. Results of Mortar Evaluation

#### 4.1.1. Flow of Mortar

**Figure 4:**
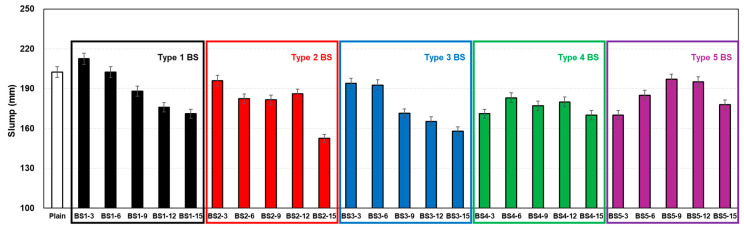
Slump of fresh mortar.

Figure 4 presents the relationship between the slump values and modified BS types and contents. The notation ‘BSx-y’ indicates BS type (x) and content (y). The plain mixture (OPC) exhibited a slump of approximately 203 mm, with most specimens showing lower slump values than OPC, except BS1-3 and BS1-6. BS types 1–3 exhibited a trend of decreasing slump values as the BS content increased. BS type 4 maintained slump values in the range of 170–183 mm, showing relative stability rather than a gradual decrease with increasing BS content. BS type 5 demonstrated increasing slump values of up to approximately 197 mm with an increasing replacement ratio, before decreasing to approximately 178 mm at 15% replacement. The modified BS types were determined based on limestone ratios at constant BS contents. BS type 4 demonstrated the most stable workability characteristics with a BS-to-limestone = 1:1 ratio, showing minimal slump variations with an increasing replacement ratio. BS types 1–3, containing higher limestone ratios than BS type 4, showed progressive slump reductions with an increasing replacement ratio, which was attributed to the effects of unmelted BS and limestone [31]. BS type 5 exhibited comparatively higher slump values owing to its lower limestone content relative to BS type 4.

#### 4.1.2. Compressive Strength of Mortar

**Figure 5:**
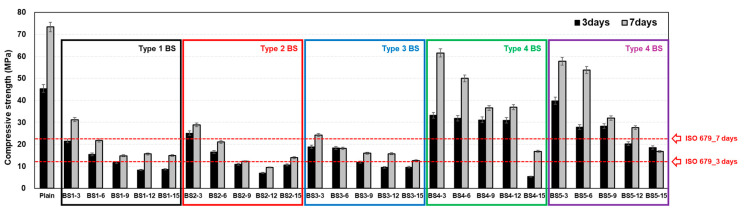
Compressive strength of mortar.

Figure 5 shows the compressive strength results based on the modified BS type and content. Specimens containing 3% of any type of BS, along with OPC, satisfied the ISO strength requirements for both 3 and 7 d. However, the BS type 1–3 mixtures with 6–15% contents failed to meet ISO specifications. All specimens of BS types 4 and 5 met ISO specifications, with the exception of those containing 15% BS. At 7 d, 3% content specimens of BS types 4 and 5 demonstrated peak strengths of approximately 61.55 and 57.55 MPa, respectively. However, these results remained below the 73.35 MPa achieved by OPC, with the strengths showing a gradual decline as the BS content increased.

**Figure 6:**
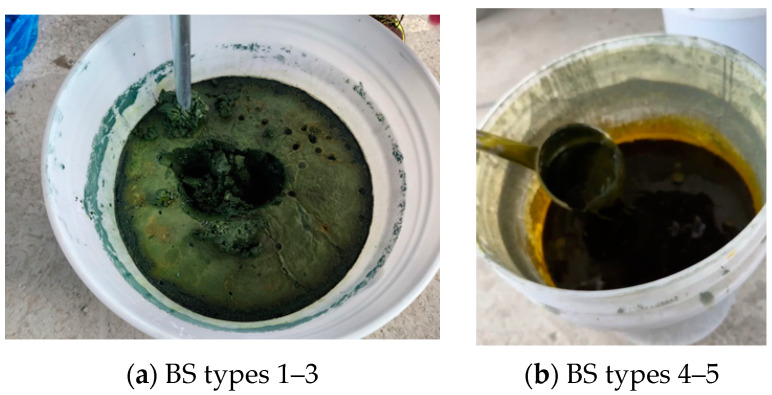
Compressive strength of mortar.

Figure 6 presents the modified BS conditions relative to the limestone content. BS types 1–3, with sulfur-to-limestone ratios of 1:1.5 to 1:5, transitioned into a solid form, as shown in Figure 6a, through moisture absorption after modification. In contrast, BS types 4–5, with sulfur-to-limestone ratios below 1:1, maintained their slurry form, as shown in Figure 6b. When mixing with the solid but imperfectly powdered form shown in Figure 6a, the BS achieved significantly lower strength, likely owing to matrix distribution issues and agglomeration. However, BS types 4–5 maintained their slurry forms, likely facilitating more uniform BS distributions throughout the mortar matrix, thus contributing to higher strength development.

In evaluating material stability and quality related to the slurry form maintenance of modified BS, BS type 4 or BS type 5 appear most suitable. Based on the comprehensive mortar slump and compressive strength evaluations, BS type 4 was ultimately selected for concrete mixing experiments, justified by its stable slump characteristics with increasing content and superior compressive strength compared to BS type 5.

### 4.2. Results of Concrete Evaluation

#### 4.2.1. Slump, Flow and Unit Weight of Concrete

**Figure 7:**
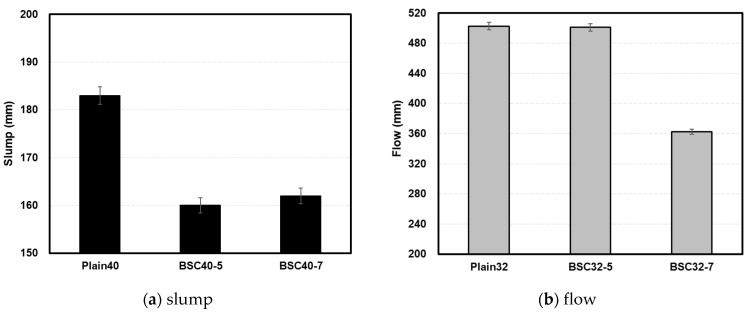
Slump and flow of fresh concrete.

Figure 7 presents the slump and flow measurements of fresh concrete. Concrete specimens with W/B ratios of 0.40 and 0.32 were classified as normal and high strength, respectively. Accordingly, the workability measurements of fresh concrete were categorized into slump and flow.

Plain40 exhibited a slump of 183 mm, whereas BSC40-5 and BSC40-7 showed lower values of 160 and 162 mm, respectively, attributed to the presence of sulfur and limestone in the modified BS. Plain32 demonstrated a flow of 503 mm, with BSC32-5 showing a comparable performance at 501 mm. However, BSC32-7 showed a notably lower value of 363 mm. Although previous research [32,33] noted that sulfur neither enhances nor decreases fresh concrete workability because of its lower specific gravity, higher fineness, and lower absorption rate than cement, the high absorption rate of limestone in the modified BS appeared to reduce workability as the content increased.

**Figure 8:**
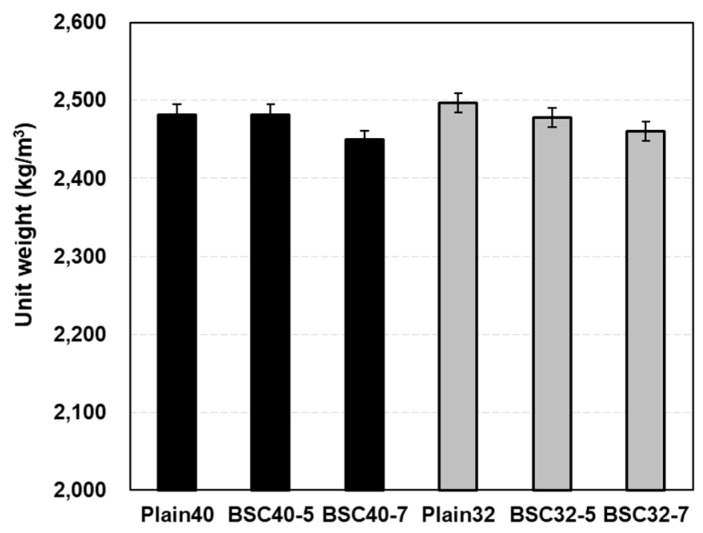
28 d unit weight of concrete.

Figure 8 illustrates the 28 d unit weight measurements relative to the modified BS content. Plain40 showed a unit weight of 2482.15 kg/m^3^, with BSC40-5 displaying nearly identical values. BSC40-5 measured 2449.33 kg/m^3^, which is 1.32% lower than Plain40, indicating minimal variations in the 0.40 W/B concrete. Plain32 reached 2496.94 kg/m^3^, exceeding Plain40 by 14.80 kg/m^3^ due to its higher cement content. BSC32-5 measured 2478.29 kg/m^3^, while BSC32-7 measured 2460.91 kg/m^3^, representing a 1.44% reduction compared to Plain32. Despite sulfur’s lower density (1.73 kg/m^3^ less than cement), the limestone addition in the modified BS resulted in minimal unit weight variations.

#### 4.2.2. Compressive Strength of Concrete

**Figure 9:**
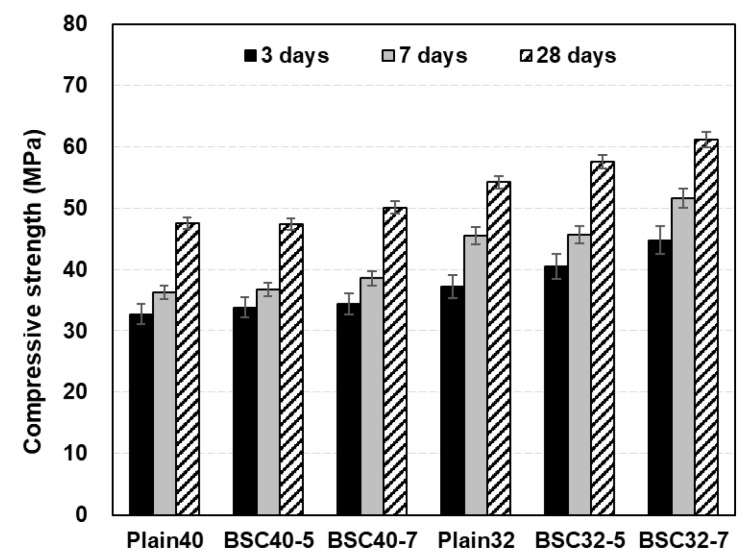
Compressive strength of concrete according to BS admixture rate.

Figure 9 shows the compressive strength development of the concrete according to age and modified BS content. All specimens reached the target strength at 28 d, showing strength increases with higher modified BS contents. For the 0.4 W/B concrete specimens, Plain40, MBS40-5, and MBS40-7 exhibited similar strength development, with BSC40-7 exceeding Plain40 by only 2.54 MPa.

In contrast, the 0.32 W/B concrete specimens demonstrated more pronounced strength increases with BS than the 0.40 W/B specimens. At 3 d, BS32-5 and BS32-7 surpassed Plain32 by 8.96% and 20.41%, respectively. Previous research [34] noted early hydration reactions and enhanced strength development with sulfur replacing the cement, which is consistent with this study’s observations of higher early strength compared to all Plain specimens. By 7 d, the strength differentials of BS32-5 and BS32-7 decreased to 0.22% and 13.22%, respectively, compared to Plain32. At 28 days, BS32-5 and BS32-7 maintained 6.20% and 12.90% higher strengths than Plain32. Earlier studies [34,35,36] with petroleum sulfur and powder sulfur as cement replacements attributed the strength gains to sulfur’s void-filling effect, which enhanced ettringite formation and CaSO4 production from the sulfur, cement and calcium reactions. The limestone and modified slurry admixture in this study similarly contributed to strength enhancement, and a detailed chemical product analysis is presented in Section 4.3.

#### 4.2.3. Ultrasonic Pulse Velocity of Concrete

**Figure 10:**
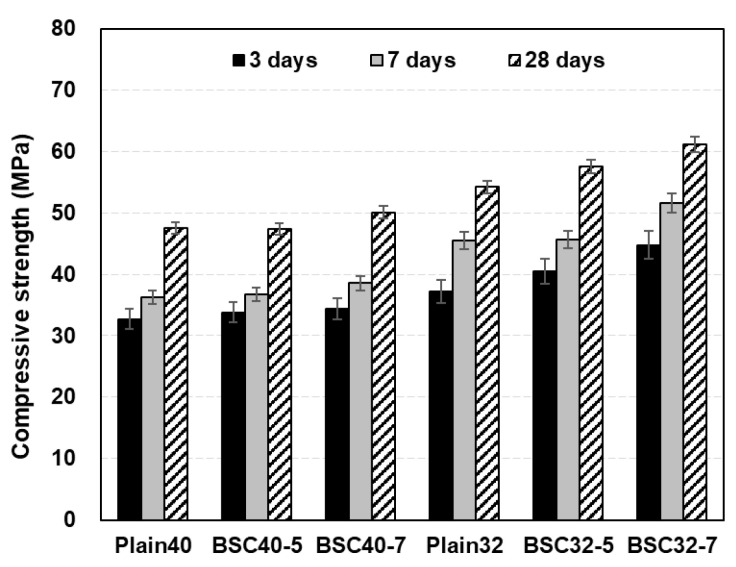
UPV of concrete according to BS admixture rate.

Figure 10 presents the UPV measurement data relative to specimen age and modified BS content. After 28 d, the UPV values for all concrete specimens were 4500 m/s or faster, achieving an ‘Excellent’ classification according to established UPV-based concrete quality criteria (Table 5) [37,38].

Plain40 and BSC40-5 demonstrated nearly identical UPV values of approximately 4773 m/s, with BSC40-7 reaching 4804 m/s. For concrete specimens with a 0.32 W/B ratio, the UPV measurements increased proportionally with the modified BS content (Plain32: 4766 m/s, BSC32-5: 4790 m/s, and BSC32-7: 4829 m/s). Although the UPV values exhibited patterns similar to the compressive strength as the modified BS content increased, no notable variations were observed between the different W/B ratios. Several previous studies have investigated UPV applications for concrete strength prediction.

However, ultrasonic waves do not maintain a direct correlation with concrete strength, as they primarily respond to medium elasticity and are significantly influenced by matrix cracks, void presence, and aggregate–mortar interfaces. High UPV measurements serve as indirect indicators of matrix density and the presence of minimal voids or cracks. Thus, while varying W/B ratios and BS content (with its lower density compared to cement) may affect matrix void structure and elasticity, these mixture proportions do not appear to significantly influence UPV progression.

### 4.3. Results of XRD

**Figure 11:**
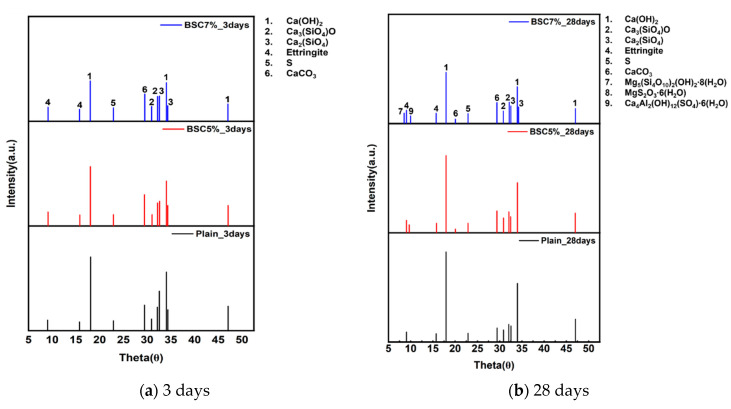
XRD analysis of concrete.

Figure 11 shows the XRD analysis results at 3 and 28 d. Crystalline phase peaks identified in all Plain specimens can be consistently observed in the modified BS specimens. While portlandite (Ca(OH)_2_) peaks can be detected at both 3 and 28 d, they exhibit diminishing intensity with increased modified BS contents. At 28 d, the 7% modified BS specimens exhibit Palygorskite (Mg_5_(Si_4_O_10_)_2_(OH)_2_·8(H_2_O)) peaks. Additionally, Kuzelite (Ca_4_A_l2_(OH)_12_(SO_4_)·6(H_2_O)) peaks emerge in the modified BS specimens at 28 d. BS, as an alkaline hydrophilic sulfur compound, appears to promote Mg ion dissolution from cement when present in sufficient quantities over extended curing periods, leading to Mg–S hydrate formation. Further, the presence of Ca–Al–S hydrates is indicated. This suggests that while initial hydration up to 3 d primarily produces cement hydrates, prolonged aging leads to the formation of complex compounds influenced by the presence of BS.

### 4.4. Results of Regression Analysis

Numerous researchers have examined UPV applications in concrete strength prediction, frequently mentioning its utility for quality assessment during maintenance phases [39,40]. Traditional studies have performed regression analysis between compressive strength and UPV values at specific curing ages. However, this approach assumes a direct correlation between concrete compressive strength and UPV measurements. Concrete in maintenance-phase structures has completed hydration, with deterioration primarily occurring through carbonation, fire exposure, and crack formation. According to conventional research methods, as concrete undergoes active hydration, the matrix structure remains incomplete and contains a relatively high water content. Our analysis of heat-deteriorated concrete strength prediction models revealed that heat-exposed concrete exhibits markedly reduced UPV values compared to specimens of equivalent strength [41]. Mehmandari et al. [42] evaluated UPV at varying load levels (20%, 40%, 60%, and 80% of ultimate failure load) and demonstrated significantly lower UPV measurements than previous findings. This sensitivity to deterioration stems from UPV’s susceptibility to scattering, reflection, and refraction effects from cracks and voids [43].

Therefore, UPV measurements by concrete age appear most suitable for determining precise formwork removal times during construction phases. Researchers have previously proposed UPV applications for cement setting-time evaluation, while Nam et al. [44] specifically addressed vertical formwork removal time assessment using UPV. Our study presents UPV values corresponding to minimum formwork removal strengths specified by ACI 347R and BS 8010. While ACI 347R omits specific vertical formwork removal strength requirements, it mandates horizontal formwork removal strength exceeding 70% of design compressive strength. BS 8010 establishes a minimum 10 MPa threshold for both vertical and horizontal formwork removal.

**Figure 12:**
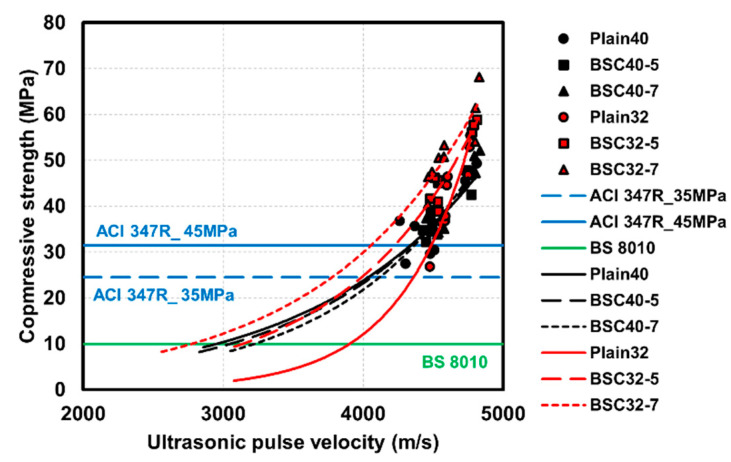
Regression analysis between compressive strength and UPV on concrete.

Table 6 presents the regression analysis results, with Figure 12 providing a visualization of the regression models in comparison to ACI 314R [45] and BS 8010 standards. The regression models employ exponential functions, consistent with both our previous research and established studies on age-based concrete behavior. Statistical analysis revealed *p*-values below the 0.05 significance threshold for all specimens, confirming a meaningful correlation between UPV (independent variable) and compressive strength (dependent variable). Moreover, R-square (R^2^) values ranging from 0.70 to 0.89 demonstrated robust model reliability. Table 7 summarizes UPV values corresponding to formwork removal strengths specified in ACI 347R and BS 8010.

## 5. Conclusions

In this study, evaluations of modified BS-incorporated mortars and concrete were conducted, with the key findings summarized below.

(1) With increasing BS content, mortars containing BS types 1–3 (sulfur to limestone = 1:1.5–5) exhibited progressive slump reduction owing to the influence of reduction due to the high absorption rate of limestone, whereas mortars incorporating BS types 4–5 (sulfur to limestone = 1:0.5–1) demonstrated consistent workability regardless of the content increase.

(2) The compressive strength of the BS type 1–3 mortars showed a 57.50–87.04% reduction compared to all Plain specimens, which was attributed to the influence of limestone with its high-water absorption characteristics, which caused the sulfur and limestone to solidify and prevented the BS from being evenly distributed within the matrix. BS type 4–5 mortars exhibited 16.09–77.23% lower strength at 3% BS incorporation, with optimal incorporation rates below 9% for BS types 4–5.

(3) Finally, BS type 4 was selected with 5% and 7% cement replacements for the concrete mixtures. Fresh concrete with a 0.40 W/B ratio exhibited a 20 mm lower slump in all BSCs than all Plain specimens due to the higher absorption of limestone and sulfur and the effect of material consolidation, while the high-strength concrete (0.32 W/B ratio) showed comparable flow values at 5% BS content but a 140 mm reduction at 7% content.

(4) Plain40 and BSC40-5 demonstrated comparable compressive strengths of approximately 47 MPa, with BSC40-7 achieving 2.54 MPa higher strength. The high-strength concrete exhibited progressive strength increases with higher BS contents, which was attributed to the combined effects of sulfur and limestone filler action, enhancing early-age ettringite formation and CaSO_4_ production from sulfur cement–calcium interactions.

(5) The UPV measurements increased proportionally with BS content, although no significant variations were observed across the different W/B ratios. Statistical analysis revealed *p*-values below the 0.05 threshold for all specimens, confirming statistical significance. UPV ranges for formwork removal timing were 4003–4406 m/s according to ACI 347R and 2761–3832 m/s according to BS 8010 specifications.

(6) The XRD analysis solely revealed cement hydrate peaks during initial hydration (up to 3 d), whereas the 28 d specimens containing modified BS exhibited distinct peaks for Palygorskite (Mg_5_(Si_4_O_10_)_2_(OH)_2_·8(H_2_O)) and Kuzelite (Ca_4_A_l2_(OH)_12_(SO_4_)·6(H_2_O)). We assumed that extended curing periods facilitated Mg ion leaching from the cement, resulting in the formation of Mg–S hydrates and Ca–Al–S hydrate compounds.

The reaction of bio-sulfur and lime generates ettringite, which can densify cement hardener organization and reduce the penetration and diffusion of harmful ions from the outside. In addition, it is believed that unreacted calcium–sulfur compounds exist in the pores inside the cement hardeners to reduce the penetration and diffusion of harmful ions from the outside, thereby improving the durability of marine concrete. Therefore, it is necessary to conduct future tests on the durability of neutralization, ion permeation, freeze-melting, and marine exposure to consider its use as marine concrete.

## Figures and Tables

**Table 1:** Experimental plan.

Classification		Program
Specimen dimension		Mortar (400 × 400 × 1600 mm), Concrete (Φ 100 × 200 mm)
Cement		Ordinary Portland cement (Type I)
Mineral admixture (mortar)		Bio-sulfur (0%, 3%, 6%, 9%, 12%, 15%)
Mineral admixture (concrete)		Bio-sulfur (0%, 5%, 7%)
Bio-sulfur types	BS1	Sulfur/limestone = 1:1.5
	BS2	Sulfur/limestone = 1:3
	BS3	Sulfur/limestone = 1:5
	BS4	Sulfur/limestone = 1:1
	BS5	Sulfur/limestone = 1:0.5
W/B ratio		0.40, 0.32
Curing conditions		Water, Room temperature (20 ± 2 °C)
Curing		Mortar: 3, 7 days Concrete: 3, 7, 28 days
Test items		Slump, Unit weight, Compressive strength, Ultrasonic pulse velocity, XRD, Regression analysis

**Table 2:** Physical properties of the materials.

Materials	Properties
Cement	Type I Ordinary Portland cement Density: 3.14 g/cm^3^, Fineness: 3200 cm^2^/g
Bio-sulfur	Bio-sulfur Density: 1.41 g/cm^3^, Fineness: 3900 cm^2^/g
Limestone	Limestone Density: 2.80 g/cm^3^, Fineness: 4500~5000 cm^2^/g
Coarse aggregate	Crushed granite aggregate Density: 2.70 g/cm^3^, Absorption: 1.0%
Fine aggregate	River sand Density: 2.63 g/cm^3^, Absorption: 1.3%
Super plasticized	Polycarboxylic-based acid

**Table 3:** Chemical properties of binder.

Materials	Chemical Composition (%)						
	CaO	SiO_2_	Al_2_O_3_	Fe_2_O_3_	MgO	SO_3_	K_2_O
OPC ^1^	60.34	19.82	4.85	3.30	3.83	2.88	1.08
BS ^2^	27.62	0.36	0.17	0.09	1.32	69.36	0.03

^1^ Ordinary Portland Cement; ^2^ Bio-sulfur.

**Table 4:** Mix proportions of concrete.

ID	W/B	S/a (%)	Unit Weight (kg/m^3^)				
			W	C	MBS	S	G
Plain40	0.40	46.0	160	400	-	814	981
BSC40-5				380	20	804	969
BSC40-7				372	28	800	965
Plain32	0.32			500	-	775	934
BSC32-5				475	25	763	920
BSC32-7				465	35	759	914

**Table 5:** Concrete quality according to UPV.

Quality	Velocity
Excellent	4500 m/s or faster
Good	3500–4500 m/s
Medium	3000–3500 m/s
Doubtful	2000–3000 m/s
Very weak	2000 m/s or slower

**Table 6:** Regression analysis results.

ID	Formula	a	b	R-Square (R^2^)	RMSE	*p*-Value
Plain40	y = a*eb*UPV	0.8631	0.0008	0.70	5.55	<0.0001
BSC40-5		0.6678	0.0009	0.83	2.67	<0.0001
BSC40-7		0.3952	0.0010	0.83	2.79	<0.0001
Plain32		0.0047	0.0020	0.80	7.76	<0.0001
BSC32-5		0.3633	0.0011	0.89	10.80	<0.0001
BSC32-7		0.8335	0.0009	0.78	2.57	<0.0001

**Table 7:** UPV at demolding standard compressive strength.

Standard	Plain40	BSC40-5	BSC40-7	Plain32	BSC32-5	BSC32-7
ACI 347R	4183 m/s	4003 m/s	4127 m/s	4406 m/s	4057 m/s	4036 m/s
BS 8010	3062	3007	3231	3832	3014	2761

## Data Availability

The original contributions presented in the study are included in the article; further inquiries can be directed to the corresponding author.
